# *Brachypodium distachyon*: a new pathosystem to study Fusarium head blight and other *Fusarium *diseases of wheat

**DOI:** 10.1186/1471-2229-11-100

**Published:** 2011-06-03

**Authors:** Antoine Peraldi, Giovanni Beccari, Andrew Steed, Paul Nicholson

**Affiliations:** 1 Department of Disease and Stress Biology, John Innes Centre, Colney Lane, Norwich, NR4 7UH, UK; 2 Dipartimento di Scienze Agrarie e Ambientali, Facoltà di Agraria, Università degli Studi di Perugia, Borgo XX Giugno 74, Perugia, 06121, Italy

**Keywords:** *Fusarium*, *Brachypodium distachyon*, wheat, deoxynivalenol, model-to-crop translation, disease resistance, host-pathogen interaction

## Abstract

**Background:**

*Fusarium *species cause Fusarium head blight (FHB) and other important diseases of cereals. The causal agents produce trichothecene mycotoxins such as deoxynivalenol (DON). The dicotyledonous model species *Arabidopsis thaliana *has been used to study *Fusarium*-host interactions but it is not ideal for model-to-crop translation. *Brachypodium distachyon *(Bd) has been proposed as a new monocotyledonous model species for functional genomic studies in grass species. This study aims to assess the interaction between the most prevalent FHB-causing *Fusarium *species and Bd in order to develop and exploit Bd as a genetic model for FHB and other *Fusarium *diseases of wheat.

**Results:**

The ability of *Fusarium graminearum *and *Fusarium culmorum *to infect a range of Bd tissues was examined in various bioassays which showed that both species can infect all Bd tissues examined, including intact foliar tissues. DON accumulated in infected spike tissues at levels similar to those of infected wheat spikes. Histological studies revealed details of infection, colonisation and host response and indicate that hair cells are important sites of infection. Susceptibility to *Fusarium *and DON was assessed in two Bd ecotypes and revealed variation in resistance between ecotypes.

**Conclusions:**

Bd exhibits characteristics of susceptibility highly similar to those of wheat, including susceptibility to spread of disease in the spikelets. Bd is the first reported plant species to allow successful infection on intact foliar tissues by FHB-causing *Fusarium *species. DON appears to function as a virulence factor in Bd as it does in wheat. Bd is proposed as a valuable model for undertaking studies of Fusarium head blight and other *Fusarium *diseases of wheat.

## Background

Several *Fusarium *species are globally important pathogens of wheat (*Triticum aestivum*). These fungi infect floral tissues as well as seedlings, stem bases and roots causing Fusarium head blight (FHB), seedling blight, crown rot and root rot, respectively [1,2]. Of these, FHB is the one of greatest significance worldwide being one of the most destructive diseases of wheat, with economic and health impacts [3,4]. The predominant *Fusarium *species associated with FHB are *Fusarium graminearum *(Fg) (teleomorph: *Gibberella zeae*) and *Fusarium culmorum *(Fc) which are also the most economically relevant [5,3].

FHB is of primary concern because Fg and Fc produce a number of secondary metabolites within infected grain that are toxic to human and animal consumers. The most prevalent *Fusarium *mycotoxins in wheat are trichothecenes such as deoxynivalenol (DON) and nivalenol (NIV) [6]. Experiments using mutants of Fg unable to produce DON showed that this mycotoxin functions as a virulence factor in wheat, enhancing spread of the disease within heads but in contrast plays no discernable role in barley [7]. Studies on trichothecene toxicity indicate that DON inhibits protein synthesis by binding to the 60S ribosomal subunit, activating a cellular signalling pathway resulting in a form of programmed cell death [8,9]. The phytotoxic effects of DON in wheat are chlorosis, necrosis and wilting, often leading to the bleaching of the whole head above the inoculation point [10].

The use of resistant wheat cultivars is considered to be the most effective strategy to prevent FHB epidemics and contamination of grain with trichothecenes [11]. FHB resistance in wheat has been broadly classified into two different types: resistance to initial penetration (type I) and resistance to pathogen spread within the head (type II) [12]. However, other types of resistance have also been proposed; resistance to kernel infection (type III), tolerance against FHB and trichothecenes (type IV) [13] and tolerance to trichothecene accumulation (type V) by two means: chemical modification of trichothecenes (type V-1) and inhibition of trichothecene synthesis (type V-2) [14]. Over a hundred quantitative trait loci (QTL) for FHB resistance in wheat have been reliably identified [11], but to date, only four loci have been shown to exhibit Mendelian inheritance [15-18]. *Fhb1*, derived from the resistant Chinese cultivar 'Sumai-3' is the only locus for which a molecular mechanism has been proposed. Wheat lines containing this QTL are able to convert DON into less phytotoxic DON-3-O-glycoside (type V-1) indicating that *Fhb1 *is either encoding a DON-glycosyltransferase or a modulator of the expression or activity of such an enzyme [10].

Wheat is not readily amenable for undertaking genetic studies of complex traits because of its large allohexaploid genome (three ancestral genomes totalling about 17,000 Mbp) which greatly hinders the complete genetic characterization of FHB-resistance QTLs. Because of the inherent difficulties associated with wheat, a number of alternative hosts have been proposed as models with which to investigate host-pathogen interactions in FHB. Although its genome is not yet fully sequenced, barley (*Hordeum vulgare*) presents the advantage of having a diploid genome, whilst also being a monocotyledonous plant naturally infected by *Fusarium *spp. However, barley has an inherent FHB-type II resistance [3] which can be a hindrance for studying the mechanisms underlying FHB-resistance in wheat. Rice (*Oryza sativa*) was the first monocotyledonous plant to have its genome sequenced and is a natural host for *Fusarium *spp. However, certain characteristics of rice and its interaction with *Fusarium *fungi reduce its potential for modelling FHB of wheat: rice is a tropical plant adapted to an aquatic environment at an early stage of development and is predominantly infected by *Fusarium *spp. other than those that cause FHB of wheat [19].

Several researchers have used the best studied plant model available, *Arabidopsis thaliana*, because it is ideally suited to laboratory studies and there are extensive genetic and genomic resources available [20]. Floral and foliar bioassays have been reported for studies of the interaction between Fg and Fc with Arabidopsis [21,22]. Such assays have demonstrated that NPR1 and EDS11 contribute to resistance of Arabidopsis against Fc [23] and that over-expression of the GLK transcriptional activator confers resistance to Fg [24]. However, to date, translation of findings on the genetic mechanisms involved in host resistance to *Fusarium *infection from Arabidopsis to cereal crops is scarce. One example is Chen *et al. *[25] who demonstrated that Fg exploits the ethylene (ET) signalling pathway to colonise Arabidopsis and showed that ET signalling also contributes to susceptibility of wheat to FHB. Despite the numerous advantages of using Arabidopsis as a model for FHB, it is not a natural host of *Fusarium*, and it displays different floral symptoms to those that occur on wheat [21]. Consequently, the identification of a model, genetically tractable, monocot system that is more closely related to wheat is highly desirable.

*Brachypodium distachyon *(Bd) is a temperate monocotyledonous plant of the grass family which has been proposed as a new model species for functional genomics in grasses [26]. The inbred line Bd21 has been recently sequenced to an 8 fold coverage [27]. Several aspects of Bd make it a very attractive model for temperate small grain cereals, including wheat. Bd has one of the smallest genomes found in grasses [28] comprising 5 chromosomes spanning over 272 Mbp in which about 25,000 protein-coding sequences are predicted [27]. Bd diverged just prior to the clade of the 'core pooid' genera that contain the majority of the temperate cereals, including wheat, making it potentially useful for functional genomics [26]. There is extensive chromosomal synteny between Bd and other cereals with the strongest syntenic relationship being with wheat for which about 77% of Bd genes have strong Triticeae EST matches [28]. In addition, it is possible to obtain genetic/physical locations in the wheat genome directly using Bd markers as demonstrated in the fine mapping of the complex *Ph1 *locus region in wheat [29]. A further advantage of Bd is that it is a self-fertile, inbreeding annual with a rapid life cycle of around 8-10 weeks [26] depending on the environmental growth conditions. In addition, this species is small in size (approximately 30 cm at maturity) and has undemanding growth requirements. Furthermore, resources are being developed to permit functional genetic studies to be undertaken in Bd. Several mutant collections exist including EMS and T-DNA insertional mutants [http://brachypodium.pw.usda.gov, BrachyTAG.org, 30], as well as a segregating population using Bd21 and Bd3-1 as parental lines [http://www.modelcrop.org].

The current study aims to examine the potential of Bd as a model to study interactions with *Fusarium *species and a base from which to undertake model to crop translational investigations.

## Results

### Floral infection

**Figure 1:**
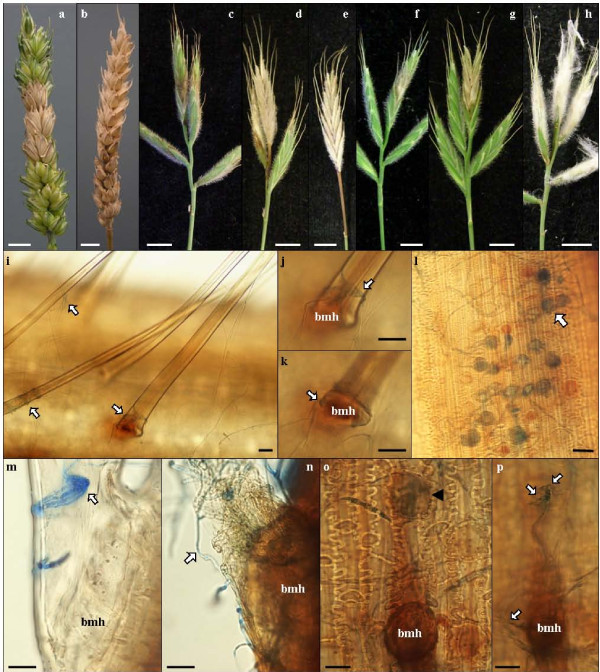
**Fusarium head blight symptoms and penetration sites on Bd spikes**. a) Typical early FHB symptoms on point inoculated wheat spike. b) Typical late FHB symptoms on point inoculated wheat spike displaying bleaching. c - e) FgUK1 spray inoculation symptoms: 3, 7 and 14 dpi, respectively. f & g) FgUK1 point inoculation, same spike 2 and 4 dpi, respectively. h) FgUK1 symptoms following spray inoculation with maintained high humidity. Scale bars a-h = 1 cm. i-p) Light microscope images of detached Bd21 florets, 3dpi with Fg, cleared and stained with aniline blue. i) External surface of lemma showing hyphal contact on macro-hairs (arrows). j & k) are close ups of picture i) taken at different focal planes. j) shows hyphal strands enveloping the macro-hair and k) shows a globose fungal structure formed at the base of the macro-hair (bmh). l) Internal surface of the palea showing hyphal colonization, necrosis and accumulation of phenolic compounds in corrugated circular cells (arrow). m & n) Macro-hair base of lemma at early stage of fungal colonization showing aggregated hyphal structure, n) Macro-hair base of lemma at late stage of fungal colonization showing extensive hyphal strands enveloping the base of the macro-hair, intense phenolic compound accumulation and collapse of the macro-hair. o-p) External surface of the palea showing the base of a macro-hair and neighbouring corrugated circular cell (arrow head) accumulating phenolic compounds (o) in response to hyphal contact (p), Upper arrow points at globose structure located above the corrugated circular cell and lower arrow pointing at hyphal strands in contact with the base of the macro-hair. Scale bars i-p = 20 μm.

FHB is the disease of greatest significance in wheat and, if Bd is to be useful as a model, it is imperative that it expresses symptoms similar to those of wheat. Spikes of Bd were spray inoculated to assess the susceptibility of Bd to Fg and Fc and to compare symptoms to those of FHB on wheat (Figure 1a,b). Optimum infection was achieved by placing plants into 8 h darkness immediately following inoculation (plants were inoculated at the start of the dark period). Similar to the situation for FHB of wheat, Bd spikes appeared to be most susceptible to infection by *Fusarium *spp at the period around mid-anthesis [4,31]. Symptom development was markedly restricted when Bd spikes were inoculated either prior to or after mid-anthesis.

Mycelial growth was detectable on the host surface from between 12 and 36 hpi and light brown, water-soaked lesions appeared proximally on the outer surface of the lemma, between 24 and 48 hpi (results not shown). From 48 h to 96 h, florets lost their green appearance and became bleached in a manner highly reminiscent of the bleaching symptoms exhibited by wheat heads with FHB (compare Figure 1a,b with Figure 1c,d). Following spray inoculation, whole spikelets became bleached and, between 96 and 144 hpi, necrotic symptoms spread down the rachis and into neighbouring spikelets above and below (Figure 1d). Disease continued to develop and between 7 and 14 days post inoculation (dpi), whole spikes became bleached and necrosis spread down into the peduncule (Figure 1e). If humidity was not maintained following inoculation, infection was reduced or even unsuccessful, leading to the total arrest of symptom development after 24 to 48 hours post inoculation (hpi), (results not shown). In contrast, maintaining high humidity for longer than 48 hpi resulted in the extensive growth of aerial mycelium which often covered the whole spike (Figure 1h). Although floral symptoms on Bd21 and Bd3-1 were similar following spray inoculation with either Fg or Fc (data not shown) disease generally developed more rapidly on Bd3-1 than on Bd21, particularly following inoculation with the Fg isolates.

Point inoculation was carried out to determine whether, like wheat, Bd exhibits susceptibility to spread within the spikelet (type II susceptibility *sensu *Schroeder and Christensen [12]). Following point inoculation, bleaching of the floral tissues tended to spread from the inoculation site towards the upper end of the spikelet with less pronounced disease progression below the point of inoculation (Figure 1f (2 dpi), 1 g (4 dpi)). Contamination of wheat grain with DON is the most important aspect of FHB with respect to food safety. The ability of Fg to produce DON within Bd tissues was investigated following spray inoculation of Bd21 spikes with Fg. Very large amounts of DON were detected in infected spikes with concentrations up to 1815 mg/kg of fresh tissue when conditions were highly conducive to infection and fungal growth (Figure 1h).

Detached Bd21 florets inoculated with Fg were studied 3dpi under a light microscope to investigate the early phase of infection in regards to pathogen penetration and early host response. Adaxial (lemma) and abaxial (palea) foliar tissues were dissected and observed individually. Extensive hyphal growth and branching was observed on the external surface of the lemma, anchoring and branching on voluminous macro-hairs (Figure 1i, arrows). Closer observation suggested that hyphae coiled around the base of macro-hairs (Figure 1j, arrow) and formed globose structures (Figure 1k, arrow) the presence of which was correlated with an amber-brown discolouration of the host tissue. At early stages of interaction, hyphae formed aggregated structures around the base of macro-hairs (BMH) with little or no discolouration of the host tissues (Figure 1m). However, at late stages of interaction, extensive hyphal growth around the BMH was correlated with intense discolouration and collapse of the host tissues (Figure 1n). Similar observations were made on the external surface of the palea where globose hyphal structures were associated with BMH and nearby cells of corrugated circular shape (Figure 1o,p) and strong amber-brown discolouration. Macro-hairs are absent from the internal surface of the palea. However, amber-brown discolouration and cell death was observed among these corrugated circular cells which we interpret to be developmentally arrested hair primordia (Figure 1l).

### Foliar infection

**Figure 2:**
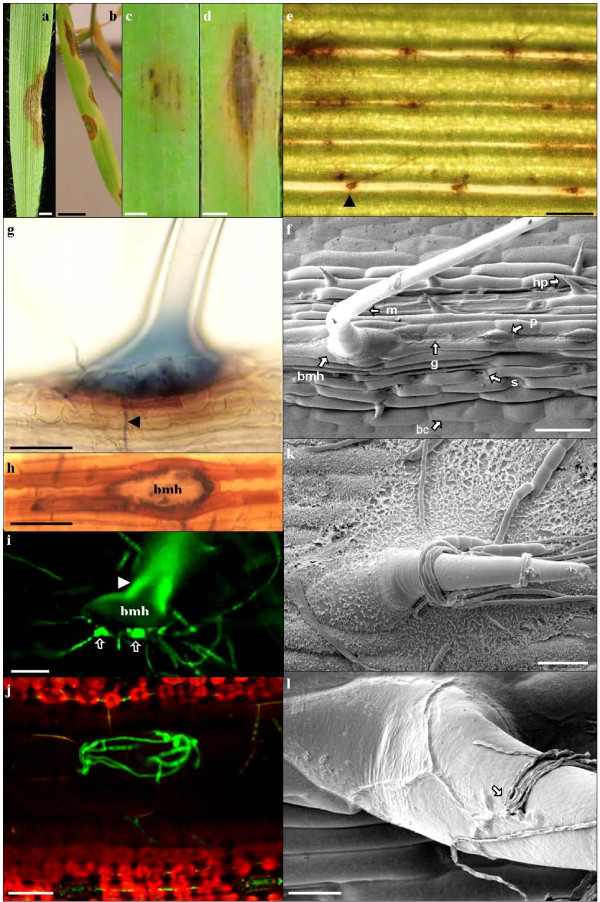
**Fusarium symptoms and penetration sites on Bd21 foliar tissue**. a & b) FgUK1symptoms on Bd21 leaves after whole plant spray. Scale bars: k = 0.5 cm, m = 1 cm: early and late symptoms, respectively. c & d) Fg symptoms on intact Bd21 detached leaf: c & e) 96hpi, and d) 144hpi. Scale bars: c & d = 0.25 cm, e = 250 μm. f) SEM image of Bd21 intact leaf surface showing Bd epidermis cell types (bc: bulliform cell, mh: macro-hair, bmh: base of macro-hair, g: girder, p: prickle cell, hp: hooked prickle, s: stomata). Scale bar = 50 μm. g and h) Light microscope images of chlorophyll cleared Bd21 leaves infected with Fg UK1, 120 hpi stained with trypan blue. Scale bars g & h = 50 μm. i) Fluorescent microscope image of Bd21 foliar macro-hair base 96hpi with GFP1-Fc. Arrow head shows macro hair endogenous fluorescence. Arrows show GFP1-Fc fluorescent hyphae forming globose structures at the bmh. Scale bar = 50 μm. j) Confocal laser scanning microscope (CLSM) image of GFP1-Fc infection on intact Bd21 detached leaf, 72 hpi, showing chlorophyll-less cells above the vascular bundles and GFP1-Fc hyphae in the cell directly beneath the bmh (bmh not in focal plane). Scale bar = 20 μm. k & l) SEM images of intact Bd21 leaf infection with FgS1, 48hpi. k) Fg hyphae enveloping a prickle cell. Scale bar = 20 μm. l) Fg hyphae aggregating near the bmh, penetrating (arrow) and growing underneath the cuticule. Scale bar = 10 μm.

Spray inoculation of whole Bd21 plants was first performed to identify tissues compatible with *Fusarium *infection. Brown, water soaked necrotic lesions developed between 48 and 72hpi on leaves (Figure 2a) followed at later stages by a surrounding chlorotic area (Figure 2b). Detached leaf assays were also performed to study symptom development on both intact and wounded foliar tissues inoculated with Fg or Fc. Following wound inoculation, dark-brown, water-soaked necrotic lesions appeared initially at the wound site between 24 and 48 hpi and extended primarily along the vascular bundles towards both the leaf tip and base (Additional file 1). Following inoculation of intact Bd foliar tissues, very small necrotic spots appeared on the leaf beneath the inoculum droplet (Figure 2c,e) followed by the appearance of more widespread necrosis. Chlorotic areas subsequently developed around these lesions (Figure 2d). Symptoms developed in a similar manner to those on the wound-inoculated leaves although progression was generally retarded by approximately 48 hours.

When studying infection processes it is important to consider the structure of the tissues. The foliar epidermis of Bd is characterised by distinct cell types organized in a succession of parallel ribs and furrows (Figure 2f). Ribs are voluminous structures which overlay the vascular bundles. They comprise different cell types organised along the longitudinal axis centred on successive wave-edged girder cells intercalated by prickle cells and voluminous macro-hairs (Figure 2f). On each side of this axis are between two and four rows of elongated cells between which lie stomata (towards the line of girder cells) and prickle cells (towards the furrow). Furrows are formed by bulliform cells.

Following inoculation onto intact leaf surfaces, Fg conidia generally aggregated in furrows. Conidia germinated between 12 and 36 hpi and hyphae grew in all directions across the leaf surface from the inoculation site. Hyphae were observed to grow towards and over stomatal apertures (results not shown) but evidence for direct penetration was not obtained.

Hyphae were frequently observed to coil around prickle cells (Figure 2k) and macro-hairs. Association with the base of macro-hairs was frequently observed (Figure 2g) and this correlated with the earliest visible host response: an amber-brown discolouration of the base of the macro-hair being particularly prominent in the cells lying immediately alongside the macro-hair (Figure 2g,h). In many instances hyphal growth was extensive about macro-hairs and globose fungal structures developed at the base of hairs (Figure 2i) and hyphae were observed with CLSM within the cell directly beneath the base of a macro-hair (Figure 2j). SEM revealed that hyphae growing on the macro-hairs could penetrate the cuticle and continue to grow beneath the cuticle towards the base of the macro-hair (Figure 2l) at which point it appears that infection proceeds, possibly via the globose structures that formed at the base of hairs (Figure 2i).

### Infection on other Bd tissues

**Figure 3:**
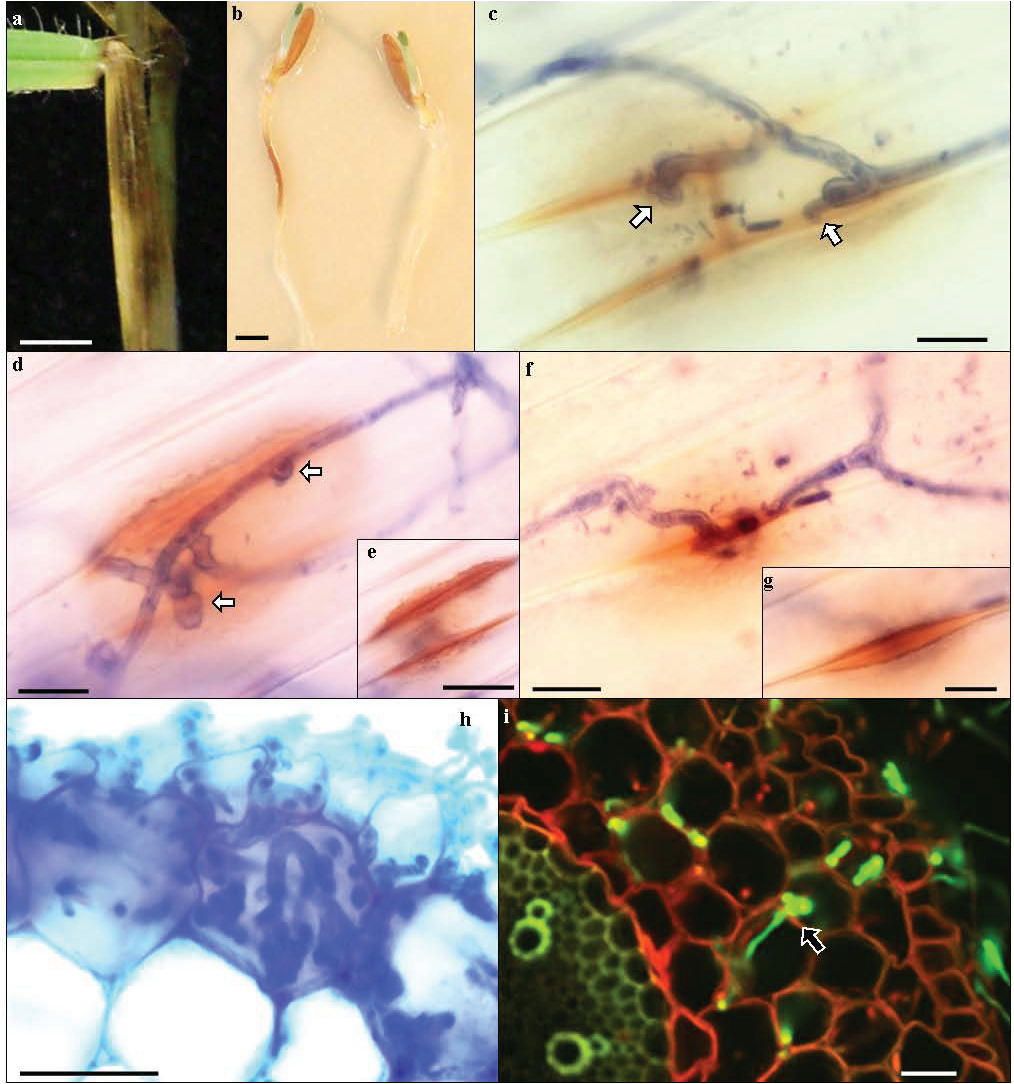
**Analysis of Fusarium infection on Bd coleoptile and root**. a) FgUK1 symptoms on leaf sheath. Scale bar = 1 cm. b) FgUK1 symptoms on Bd21 roots (left) and mock inoculation control (right), 48 hpi. Scale bar = 0.5 cm. c-g) Light microscope images of Fg UK1 infection on Bd21 coleoptiles, 6 dpi, stained with trypan blue. c) Fg hyphae penetration attempt via infection pegs (arrows) at the junction between adjacent cells showing associated deposition of phenolic compounds. d) Unsuccessful penetration attempt via infection pegs (arrows) at the junction between adjacent cells which, at lower focal plane (e), display intense deposition of phenolic compounds beneath the attempted infection point. f) Successful penetration attempt via infection pegs (arrows) at the junction between adjacent cells which, at lower focal plane (g) appear to be prised apart. Scale bars: c = 10 μm, d = 10 μm; e = 20 μm, f & g = 10 μm. h) Light microscope image of Fg UK1 at disease front of Bd21 root infection, 48 hpi stained with trypan blue. Scale bar = 20 μm. i) CLSM image of GFP-expressing Fc at infection site of Bd21 root, 48 hpi. Arrow shows hyphal translocation between two adjacent cortical cells. Scale bar = 10 μm.

Additional assays were used to investigate the ability of Fg and Fc to infect other tissues and assess the potential of Bd as a model for other cereal diseases caused by *Fusarium *species. Brown, water-soaked necrotic lesions developed between 48 and 72 hpi on virtually all above-ground plant parts including stems, stem nodes, leaf sheaths and leaves. Infected stems and stem nodes displayed only dark necrotic lesions even at late stages of the interaction (between 5 and 7 dpi) whereas necrotic areas on leaf sheaths became surrounded by chlorosis (Figure 3a).

Symptoms developed rapidly on roots of Bd21 with amber-brown discolouration present at the site of contact with the inoculum by 24 hpi (Figure 3b). Discolouration of roots continued and, from 48 hpi onwards, lesions became dark brown. Root symptoms spread in both directions along the root from the infection site until the whole root was necrotic between 96 and 120 hpi.

The outermost cell layer in the primary root of Bd is the rhizodermis, a single cell layer under which is located the cortex, made of multiple cell layers. Internal to the cortex and separated from it by the single cell layer endodermis is the stele within which lie the central metaxylem vessel and xylem vessels. Amber-brown discolouration of the roots was observed at the site of infection by 24 hpi, at which time intercellular and intracellular presence of the fungus could only be observed in the rhizodermis and the most external cortical cell layer (Figure 3h). By 48 hpi, hyphae were colonising, by both inter- and intracellular growth (Figure 3i), cortical cell layers and this was associated with the amber-brown colouration of cortical cells.

Confocal microscopy confirmed that the fungus invaded most internal layers of cortical cells by 48 hpi (Figure 3i) but hyphae were excluded from the stele even after 96 hpi (results not shown). No symptoms developed on roots following spray inoculation with Fg conidia. However, mycelium grew externally to reach the coleoptile where attempted penetration was frequently observed at the junction between adjacent cells and appeared to proceed via infection pegs (Figure 3c,d). Attempted penetration was associated with localised production of an amber-brown deposit within contacted host cells at the site of contact/attempted penetration (Figure 3d,e). In most instances fungal ingress was effectively prevented while in some cases the cells appeared to be prised apart allowing growth of the hypha between them (Figure 3f,g).

### Differential responses of Bd21 and Bd3-1 to Fg and DON

**Figure 4:**
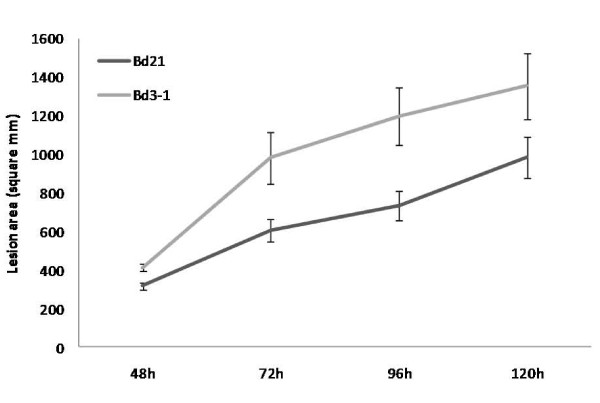
**Comparison of *Fusarium *symptoms development on Bd21 and Bd3-1 leaves inoculated with Fg**. Development of necrotic lesion area induced by Fg UK1 on wound-inoculated leaves of Bd21 and Bd3-1 at 48, 72, 96 and 120 hpi. Means ± s.e. were each calculated from measurements of twelve experimental replicates. The data shown is representative of six independent experiments.

Two Bd ecotypes, parents to a mapping population (modelcrop.org), were examined as a first step to determine the potential for natural variation for resistance to *Fusarium *within Bd. Leaves of lines Bd21 and Bd3-1 were compared for their response to wound-inoculation with Fg. Symptom development was significantly more rapid on Bd3-1 than on Bd21 (P = 0.016) (Figure 4). Most strikingly, lesions on Bd3-1 were surrounded by large areas of chlorosis whereas those on Bd21 retained their green colouration (Additional file 1). Conidial production on Bd3-1 leaves was observed to be significantly (P = 0.001) higher when compared to Bd21 leaves, 7dpi (Additional file 2).

Bd21 and Bd3-1 were also compared to assess whether they differed in type II resistance following single floret point inoculation with Fg. Disease progress as determined by AUDPC was significantly (P < 0.05) greater in Bd3-1 (31.92) than in Bd21 (20.16) (Additional file 3), although there was no significant difference in conidial production at 13 dpi, when the experiment was terminated (data not shown).

**Figure 5:**
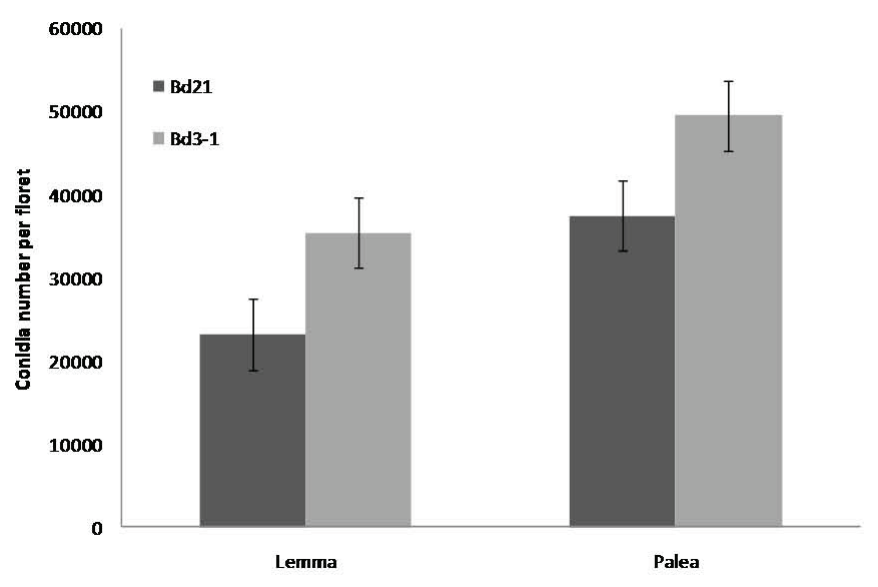
**Comparison of Fg conidial production on lemma and palea of Bd21 and Bd3-1 detached spikelets**. Conidial production following inoculation of Fg UK1 onto palea or lemma surface of Bd21 and Bd3-1 detached florets, 144 hpi. Means ± s.e. were each calculated from measurements of twenty experimental replicates. The data shown is representative of three independent experiments.

In complementary experiments, single florets of Bd21 and Bd3-1 were detached, placed on moist filter paper in Petri dishes and inoculated with conidial suspension onto either the palea or lemma surface in order to study infection of these tissues and to identify potential differences in susceptibility between the Bd lines and between the tissues. Conidial production on infected florets was significantly greater (P < 0.001) when conidia were inoculated onto the palea than onto the lemma, in both Bd21 and Bd3-1 ecotypes. In addition, conidial production on both palea and lemma was higher in Bd3-1 (49,556 and 35,400 conidia/floret, respectively) than in Bd21 (37,533 and 23,200 conidia/floret, respectively) (Figure 5).

**Figure 6:**
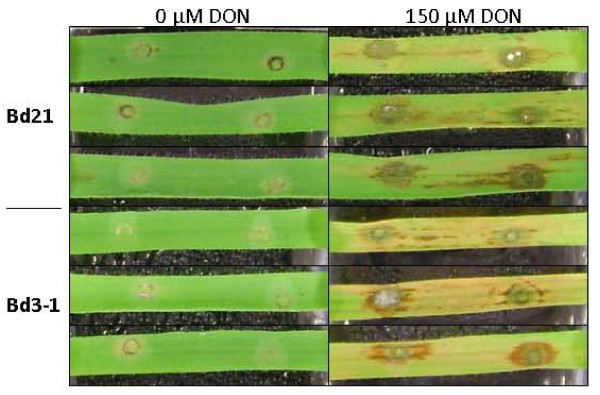
**Comparison of DON-induced lesions of Bd21 and Bd3-1 detached leaves**. Symptoms on leaves of Bd21 and Bd3-1, 120 hpi following wound-inoculation with water or DON (150 μM). Means ± s.e. were each calculated from measurements of eight experimental replicates. The data shown is representative of three independent experiments.

Lines Bd21 and Bd3-1 were also assessed for susceptibility to DON. Detached leaves were wound-inoculated with a range of DON concentrations (15, 75 and 150 μM). At the highest DON concentration, an amber-brown discolouration appeared around the wound site of both Bd 21 and Bd3-1 from 72 hpi. Lesions spread along the vascular bundles, becoming necrotic around 96 hpi. Lower DON concentrations did not result in the spread of necrotic lesions (data not shown). The size of the necrotic areas on Bd21 and Bd3-1 were not statistically different. However, chlorosis developed on Bd3-1 at all DON concentrations, whilst none was observed on Bd21 (Figure 6).

**Figure 7:**
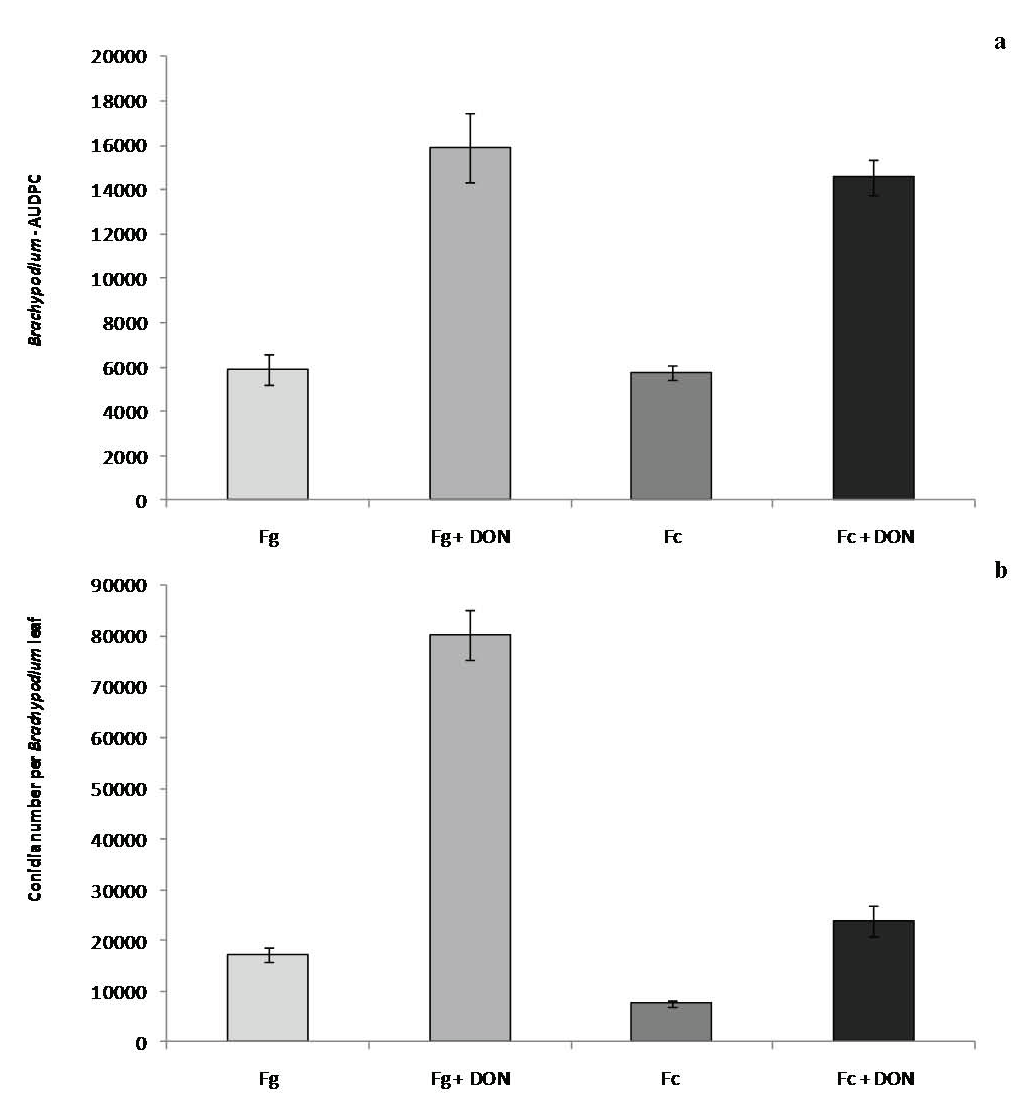
**Effect of DON treatment on Bd21 detached leaves infected with Fg or Fc**. a) Area under disease progress curve (AUDPC, 6dpi) for lesions following wound-inoculation of leaves of Bd21 with Fg UK1 or Fc GFP1 with or without amendment with DON (75 μM). b) Conidial production (6dpi) on leaves of Bd21 following wound-inoculation with Fg UK1 or Fc GFP1 with or without amendment with DON (75 μM). Means ± s.e. were each calculated from measurements of three experimental replicates. The data shown is representative of two independent experiments.

DON has been demonstrated to be a virulence factor for FHB and crown rot infection of wheat by Fg. The influence of DON on Fusarium infection of *Brachypodium *was examined on wound-inoculated detached leaves to determine whether it enhanced virulence for Fg and Fc. Amendment of conidial inoculum with DON (75 μM) significantly increased (P < 0.001) average lesion area for both Fg and Fc (Figure 7a) and conidial production (Figure 7b) when compared with infections using the conidia alone. These results were strikingly similar to the effect of DON amendment on lesion development on wheat leaves (Additional file 4).

As shown above, symptom development on floral tissues was greater in Bd3-1 than in Bd21 and additional experiments were carried out to determine whether this was also reflected in differences in accumulation of DON. Spikes of Bd21 and Bd3-1 were spray inoculated with conidia of Fg and the DON content was assessed 21 dpi. No significant difference (P = 0.971) in DON content was observed between Bd21 and Bd3-1 (620 mg/kg and 625 mg/kg of fresh tissue, respectively).

## Discussion

The present study aimed to determine the potential for Bd to act as a host to Fg and Fc and ascertain whether this interaction might serve as a model of that between *Fusarium *species and wheat. The results clearly demonstrated the compatibility of interaction between Bd and the two *Fusarium *species of greatest relevance to FHB, the major *Fusarium*-associated disease of wheat. Moreover, the development of disease symptoms closely resembled those reported in wheat.

With respect to FHB, after a short asymptomatic period, Bd spikes spray inoculated with Fg conidia displayed small brown spots, which first appeared at the middle or base of the lemma, highly reminiscent of the initial symptoms in wheat [4]. Lesions spread to infect adjacent florets, often provoking the bleaching of the upper part of the spikelet in a manner similar to that observed in wheat [32,10] and infection extended down the rachis to adjacent spikelets and even colonised peduncles as seen during infection of wheat. Overall, *Fusarium *infection of Bd spikes results in the development of symptoms that strikingly resemble those described in wheat heads infected with Fg and Fc [4].

Microscope analysis of floral tissues highlighted the potential role played by specific epidermal cell types during the early stages of infection. *Fusarium *hyphae were repeatedly observed entwined about voluminous macro-hairs that displayed a characteristic amber-brown discolouration. Globose fungal structures were repeatedly observed at the base of these hairs, suggesting that these cell types are favoured targets for penetration. Two components of resistance to FHB are widely recognised; resistance to initial infection (type I) and resistance to spread within the head (type II) [12]. The palea and lemma tissues of barley have been shown to express different levels of type I resistance with the former being more susceptible than the latter [33]. Similar differential type I susceptibility of the palea and lemma tissues of Bd was observed in the present study along with differences in type I susceptibility of the two tested inbred lines. Type II resistance is assessed by point inoculation of individual florets in wheat heads [4]. Following point inoculation of Bd florets, both Fg and Fc successfully colonised Bd spikelet tissues and spread through the rachis into neighbouring spikelets and down the peduncle, closely resembling the pattern of colonization in heads of susceptible wheat cultivars [4]. The bleaching of spikelets above the inoculation site in wheat heads is another characteristic symptom of FHB [10]. Bleaching has been correlated with the production of DON by the fungus within infected wheat heads and is also induced following injection of DON into wheat heads [10]. Our observation of bleaching of infected spikes of Bd thus resembles the situation in FHB of wheat more closely than does barley, which has an inherent type II resistance restricting *Fusarium *symptoms to the area of initial infection [3].

DON has been shown to function as a virulence factor in wheat, inhibiting the development of cell wall fortification within the rachis during FHB development [34] and aiding stem colonisation during development of crown rot [35]. In contrast DON appears to play no discernable role in disease development in heads of barley [34,7] or floral tissues of Arabidopsis [36]. Amendment of the conidial inoculum with DON significantly enhanced both disease symptoms and conidial production by Fg and Fc on wounded detached leaves of Bd. DON amendment similarly influenced symptom development and conidial production in detached wheat leaves following inoculation with Fg and Fc (Additional file 4). This strongly suggests that DON functions in Bd as it does in wheat, where it is understood to act as a virulence factor [34,35].

The detection of high concentrations of DON in Bd21 and Bd3-1 flowers following inoculation with Fg indicates that these tissues support DON production in *Fusarium *species. The levels of DON in Bd spray-inoculated spikes were similar to those reported previously following inoculation of wheat under controlled conditions [37,38]. The high levels of DON observed in floral tissues of Bd differs markedly from the situation with Arabidopsis where the reported levels are generally extremely low [23,21]. Trichothecene production has been shown not to be uniformly induced during infection of wheat but, rather, is tissue specific with induction in developing kernels and the rachis node [39]. It is probable that the necessary components to induce trichothecene production are present in Bd and wheat whereas they are absent in Arabidopsis, making Bd an attractive model for wheat. The current experiment could not provide information on kernel resistance as whole floral tissues were sampled because the high infection pressure resulted in extremely shrivelled seeds. However, reducing infection pressure and dissection of floral parts could provide insight onto resistance to kernel infection in future experiments.

Following spray inoculation of whole Bd plants, symptoms developed on virtually all above-ground plant parts (stems, leaf sheaths and leaves). Unexpectedly, intact leaves from spray inoculated plants also developed necrotic and chlorotic symptoms as did inoculated unwounded detached leaf sections. The presence of *Fusarium *within Bd tissues was confirmed by CLSM observation of GFP-expressing fungus. This is, to our knowledge, the first report to date of a successful infection on intact foliar tissue by a *Fusarium *species. Detached leaf assays have been used previously to identify components of resistance related to FHB but these experiments, although using unwounded inoculation, were carried out using *Microdochium majus*, a non-toxin producing FHB species [40]. We have determined that Fg and Fc can infect floral and foliar tissues of Bd allowing the mycotoxin-producing species to be used in comparative assays on these tissues. The susceptibility of intact Bd leaves therefore provides the first opportunity to establish the relationship between foliar and floral components of resistance to *Fusarium *species and identify those foliar components of relevance to FHB resistance. The unique susceptibility of Bd to foliar penetration by *Fusarium *spp. also provides the potential to undertake high throughput genetic screening of Bd mutant collections to identify lines altered in susceptibility to penetration. Having observed disease symptoms on all tested Bd tissues, histological examination was undertaken to determine how Fg and Fc gain entry into this host. Direct stomatal penetration of wheat head tissues by Fg and Fc has been previously reported [41-43]. Despite observing multiple instances of direct contacts between Fg and Fc germination hyphae and stomatal apertures, we did not obtain evidence for entry into Bd via stomata. Overall, our results suggest that, although penetration may occur through stomatal apertures, it is not likely to be the main mode of entry. In numerous instances, hyphal contact with stomata resulted in guard cells becoming very dark brown, indicating the possible deposition of phenolic compounds. Interestingly, phenolic compounds have been previously shown to play a role in FHB disease resistance in wheat [44] and a similar situation may occur in the guard cells of Bd. Light microscopy images of the first visible symptoms developing on leaves revealed a characteristic amber-brown discolouration (distinct from the colour of contacted guard cells), of the macro-hair base and directly adjacent cells that was correlated with the presence of the fungus and attempted penetration of the host. Although this amber-brown colour is also indicative of phenolic compounds, the results from coleoptile infection studies showed that its accumulation at the site of attempted fungal penetration is not effective in preventing infection. Similar appositions have been observed during infection of wheat by Fg and were more pronounced in resistant than in susceptible cultivars [45]. During infection of Bd coleoptiles Fg appeared to produce infection pegs and gain entry via growth between cells. Again, this is similar to infection observed on wheat [43]. SEM analysis of intact Bd leaf surface indicated that penetration of hair cells may be the preferred route of entry for the pathogen. We observed penetration of the cuticle, growth and branching at the base of the macro-hair. Macro-hairs are located above the vascular bundles, and targeting their base for initial penetration provides the pathogen almost direct access to the vascular bundles enabling rapid spread to adjacent tissues [46]. This is an interesting finding in relation to previous studies made on detached wheat glumes where Fg was observed to penetrate and invade host tissue through short hair cells (termed prickle hairs [47]). Association between Fg hyphae and prickle hairs (also referred to as papilla cells) on wheat was also noted by Pritch and colleagues [42], although they did not undertake detailed investigation of the interaction. The comparison of microscope images of infected floral and foliar Bd tissues revealed striking similarities. *Fusarium *hyphae were observed to specifically target hairs in both tissues, where globose hyphal structures developed about BMH. Accumulation of phenolic compounds of unknown composition occurred in both floral and foliar tissues as a host response to penetration attempts. These similarities support the idea that investigating the mechanisms of *Fusarium *infection on foliar tissues may have direct relevance to the mechanisms of resistance of the floral tissues to FHB.

Root tissues were also successfully infected following inoculation by contact with mycelial plugs. The infection pressure generated by conidia, however, failed to induce infection and it remains to be determined whether infection can proceed directly from conidia or whether infection requires hyphae. Infection was indicated by discolouration and confirmed by observation of inter- and intracellular fungal hyphae in the cortex at an early stage of infection. Even at late stages of infection fungal hyphae were excluded from the stele, a situation similar to that recently reported in wheat [41]. Together with observation of symptoms developing on the stem base, these results suggest that Bd can also be used for modelling crown rot and root rot.

Differential responses among Bd accessions to biotic and abiotic stresses have been observed by others indicating that naturally occurring allelic variation in Bd accessions may provide insights into mechanisms underlying responses to agronomically important traits [48,49]. Inoculation of Fg conidia on detached Bd florets revealed quantitative differences in fungal development between Bd21 and Bd3-1 lines. Interestingly, the two lines also differed in susceptibility in the detached leaf assay with the most notable difference between them being the extensive chlorosis that developed in Bd3-1. Interestingly, DON application to wounded Bd21 and Bd3-1 leaves also resulted in a difference in response with respect to the development of chlorosis indicating that the differential response of the two lines to Fg is, at least in part, a result of differential susceptibility to DON. The availability of the population derived from a cross between Bd21 and Bd3-1 (http://www.modelcrop.org), will permit genetic mapping of the differential susceptibility of these lines to DON and foliar infection. Additionally, investigating the wide range of di-, tetra- and hexaploid Bd accessions would be expected to reveal different levels and mechanisms of resistance to *Fusarium*.

Bd was previously reported as a model for rice in order to study resistance to *Magnaporthe grisea *[48]. The current study provides the first detailed report of Bd as a potential model for a wheat disease caused by a necrotrophic fungus.

## Conclusions

We demonstrate herein a compatible reaction between *Fusarium *species and Bd and establish a new pathosystem with which to investigate mechanisms underlying FHB resistance in a tractable monocotyledonous model species. Disease symptoms on Bd spikes and the accumulation of DON within floral tissues were highly similar to those on wheat heads. Futhermore, we identified naturally-occurring variation for resistance to *Fusarium *species among Bd accessions and report, for the first time, successful *Fusarium *infection of intact foliar tissues. Infection of both floral and foliar tissues were highly similar, strongly suggesting direct relevance of findings from one tissue to the other. Synteny between Bd and wheat is very high making possible the direct translation of information on the role of particular genes in resistance in Bd to their counterparts in wheat. This, taken together with the availability of a complete genome sequence and an increasing number of resources for functional genomics, gives Bd the potential to become a significant model species with which to investigate resistance to *Fusarium *species and provide information of direct relevance to wheat and other cereal crops.

## Methods

### Maintenance and preparation of *Fusarium *inoculum

DON-producing isolates of Fg (UK1 and S1) from the culture collection of the John Innes Centre were used throughout. A DON-producing constitutive GFP expressing isolate (FcGFP1) of Fc (kindly provided by Dr F. Doohan, University College Dublin, Ireland) was used for confocal microscopy. Conidial inoculum was produced in mung bean (MB) liquid medium and prepared as described previously with shaking for 7 days at 25°C [50]. To harvest conidia, the culture solution was filtered through sterilized muslin and centrifuged at 3000 g for 5 min. The pellet was washed once and re-suspended in sterile distilled water (SDW) at a concentration of 1 × 10^6 ^conidia ml^-1 ^and stored at -20°C until use.

### *Brachypodium *lines and growth conditions

*Brachypodium distachyon *inbred lines Bd21 and Bd3-1 [51] were used throughout. Bd seeds were germinated and incubated for 5 days in Petri dishes on damp filter paper in the dark at 5°C. Seed were then incubated in darkness at 15°C for 24 h before exposing to a 16 h/8 h light-dark cycle for 24 h at 20°C. Seeds were then planted in 8 × 8 × 10 cm pots filled with 50% peat and sand mixed with 50% John Innes number 2 loam compost, and placed in a climatically controlled chamber with a relative humidity (RH) of 70% at 22°C. Foliar tissue was obtained from plants grown under a 16 h/8 h light-dark cycle while plants were grown under a 20 h/4 h light-dark cycle to obtain floral tissues.

### *Brachypodium *spray, point, coleoptile and root inoculations, incubation and symptom assessment

Whole Bd21 plants were sprayed with FgUK1 conidial suspension (1 × 10^5 ^conidia ml^-1^), amended with 0.05% Tween 20, using a handheld mister until run off. Sprayed plants were placed under a plastic cover and misted periodically with SDW to increase relative RH to about 90%, until 3 days post inoculation (dpi) when covers were removed and misting ceased. Disease symptoms were photographed using a Samsung NV7 digital camera. Floral point inoculations were performed by inserting a piece (2 × 8 mm) of filter paper (Sartorius; grade 292) between two adjacent florets. Conidial suspension (5 μl of 1 × 10^6 ^conidia ml^-1^) of Fg UK1 was carefully applied to the filter paper. Following inoculation, plants were treated as for spray inoculation above. Floral symptom development was quantified by visual assessment and the number of infected florets was counted at 2, 4 and 8 dpi.

Studies on infection of roots and coleoptiles were carried out on Bd seedlings germinated as described above and incubated for 5 days at 20°C under a 16 h/8 h light-dark cycle. Seedlings used for root infection were inoculated using a mycelium plug (5 mm dia) from the growing edge of a 14 day old colony grown on potato dextrose agar (PDA) at 20°C and with a PDA plug for the controls. Coleoptiles were inoculated by spraying 1 ml of conidial suspension (1 × 10^6 ^conidia ml^-1^) per plate and with sterile water for the controls.

Bd21 and Bd3-1 flowers were harvested 21 days after spray inoculation with Fg S1 or Fc GFP1 conidial suspension (1 × 10^5 ^conidia ml^-1^), frozen in liquid N_2 _and ground to a fine powder. DON detection and quantification was performed using an ELISA competitive immunoassay (AgraQuant^®^, Romer Labs Singapore Pte Ltd) according to the manufacturer's recommendation.

### Inoculation, incubation and symptom assessment of detached leaves

Leaves were removed from 21 days old plants, cut to 5 cm length and wounded in two positions 2 cm apart and on opposite sides of the mid-rib by gentle compression with a glass Pasteur pipette on the adaxial surface. Leaf sections were placed in 10 × 10 cm square plastic boxes containing 0.8% water agar and treated as reported previously for wheat and barley [25]. Each box contained eight leaf sections from different plants. A droplet (10 μl) of conidial suspension (1 × 10^6 ^conidia ml^-1^), amended with 0.05% Tween 20, was deposited onto each wound site. In other experiments the conidial suspension was amended with DON (75 μM). Mock inoculation was performed similarly using SDW amended with 0.05% Tween 20 (10 μl).

In separate experiments, unwounded leaves were similarly inoculated with Fg or treated by addition of DON (15, 75 and 150 μM amended with 0.01% Tween 20). The inner surface of the plate lid was misted with SDW to maintain 100% RH and plates were incubated at 22°C under a 16 h/8 h light-dark cycle. Disease symptoms were recorded every 24 h and lesion sizes were measured using IMAGE-J software [52].

### Light microscopy

Bd leaf sections and flowers were cleared in 70% ethanol at 70°C for one hour to remove chlorophyll. Samples were stained for 1 min in trypan blue or aniline blue (0.1%) in lactoglycerol (1:1:1, lactic acid: glycerol: H_2_O) and rinsed in a 15 M solution of chloral hydrate. Samples were mounted in 40% glycerol, viewed with a Nikon Eclipse 800 microscope and photographed with a Pixera Pro ES 600 digital camera. Inoculated palea and lemma tissues were dissected, cleared of chlorophyll, stained with aniline blue and observed under a light microscope.

### Confocal microscopy

Horizontal cross sections (50 μm thickness) of roots (inoculated and non-inoculated) were dissected 24, 48, 72 and 96 h post inoculation (hpi) using a sectioning system (Vibratome 1000 plus) and placed between glass slides in SDW. Root sections were analysed under a confocal microscope (Leica DMR SP1) excited with a 488 nm Argon ion laser and detected at 505-555 nm. Autofluorescence of cell walls and chloroplasts was detected at 580-680 nm.

### Scanning electron microscopy

Intact Bd leaf sections were mounted on an aluminum stub by using O.C.T. compound (BDH), plunged into liquid nitrogen slush, and then transferred onto the cryostage of an ALTO 2500 cryo-transfer system (Gatan) attached to a Zeiss Supra 55 VP FEG scanning electron microscope. Samples were then sputter-coated with platinum (90 s at 10 mA, -110°C), and imaged at 3 kV on the cryo-stage in the main chamber of the microscope at -130°C. An alternative fixation method was used to remove wax crystals from the surface of Bd leaves and allow observation of sub-cuticular structures. Leaf sections were fixed for approximately 4 hrs at 20°C in FAA (3.7% formaldehyde, 5% acetic acid, 50% ethanol) and subsequently dehydrated through an ethanol series. After critical point drying, tissues were coated with gold and examined in a Philips XL30 FEG microscope using an acceleration voltage of 3 kV.

### Statistical analysis

The disease severity, conidial production, DON accumulation and lesion area data were analysed by generalised linear modelling (GLM) using the software package GENSTAT version 9.1 (Lawes Agricultural Trust, Rothamsted Experimental Station, UK). Individual treatments were compared with controls using the unpaired *t*-tests within the GLMs.

## Authors' contributions

AP carried out all Bd spray and point inoculations, disease assessments and detached leaf assays and normal light and SEM microscopy analysis. GB carried out Bd root inoculations and CLSM analysis of root tissues and participated to Bd detached leaf assays. AS and PN took part in designing and supervising the study and participated in drafting the manuscript. All authors have read and approved the final manuscript.

## Supplementary Material

Additional file 1 **Comparison of symptoms following Fg infection on Bd21 and Bd3-1 leaves**. Symptoms on leaves of Bd21 and Bd3-1, 120 h following wound inoculation with Fg UK1. Click here for file

Additional file 2 **Comparison of Fg conidial production on Bd21 and Bd3-1 detached leaves**. Conidial production following inoculation of Fg UK1 onto Bd21 and Bd3-1 detached leaves, 7 dpi. Click here for file

Additional file 3 **Comparison of necrotic symptoms development following Fg point inoculation on Bd21 and Bd3-1 spikelets**. Area under disease progress curve (AUDPC) for lesions of Bd21 and Bd3-1 spikelets point inoculated with Fg UK1. Click here for file

Additional file 4 **Effect of DON treatment on detached wheat leaves infected with Fg or Fc**. a) Area under disease progress curve (AUDPC) for lesions following wound-inoculation of wheat (cv. Paragon) leaves with Fg UK1 and Fc GFP1 with or without amendment with DON (75 μM). b) Conidial production (6dpi) on leaves of wheat (cv. Paragon) following wound-inoculation with Fg UK1 and Fc GFP1 with or without amendment with DON (75 μM). Click here for file
