# Supplementary figures and images for: Brachypodium distachyon: a new pathosystem to study Fusarium head blight and other Fusarium diseases of wheat
Source: BMC Plant Biol. 2011 Jun 3;11:100. doi: 10.1186/1471-2229-11-100 (PMC3123626; doi:10.1186/1471-2229-11-100)

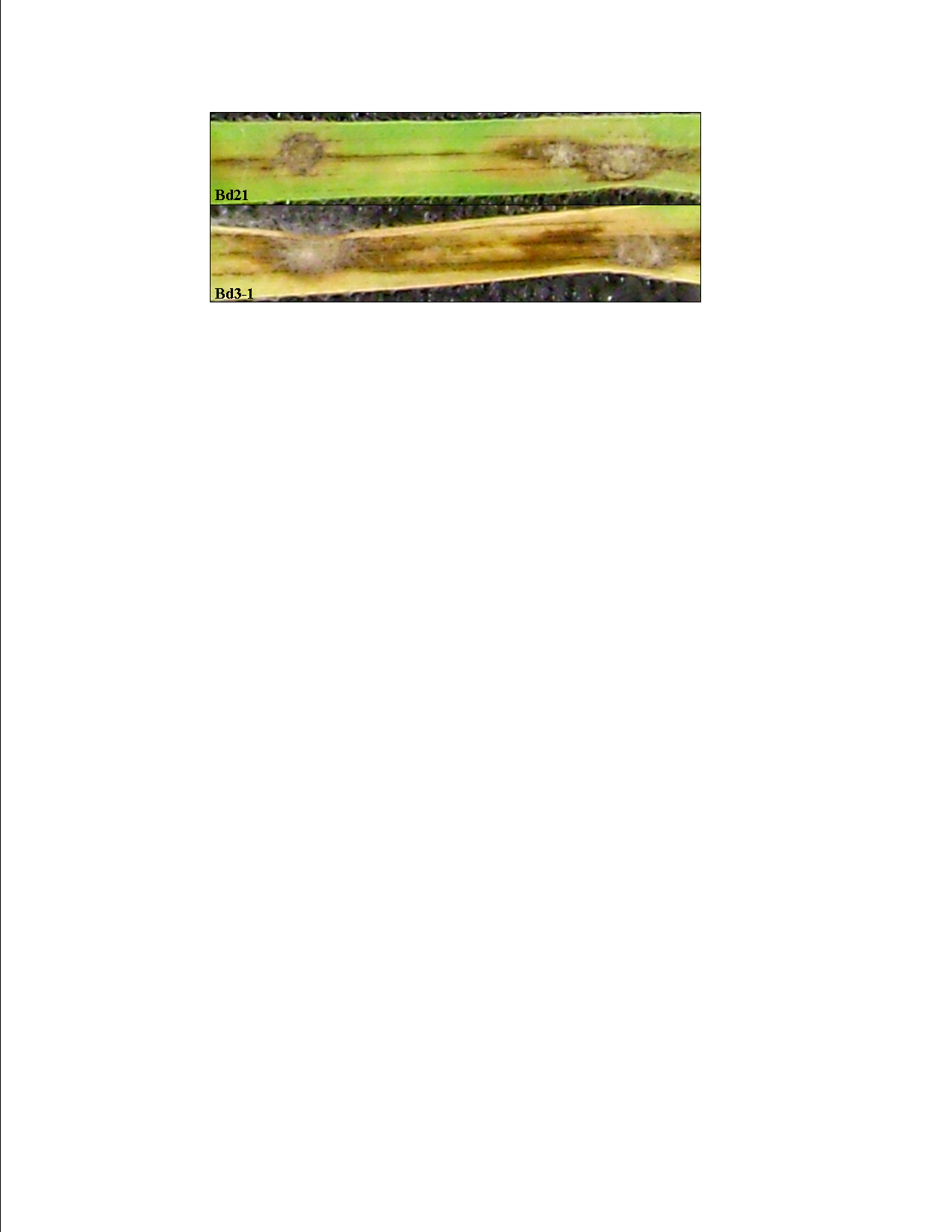

Supplement: Additional file 1 — Comparison of symptoms following Fg infection on Bd21 and Bd3-1 leaves. Symptoms on leaves of Bd21 and Bd3-1, 120 h following wound inoculation with Fg UK1. [file 1471-2229-11-100-S1.TIFF]

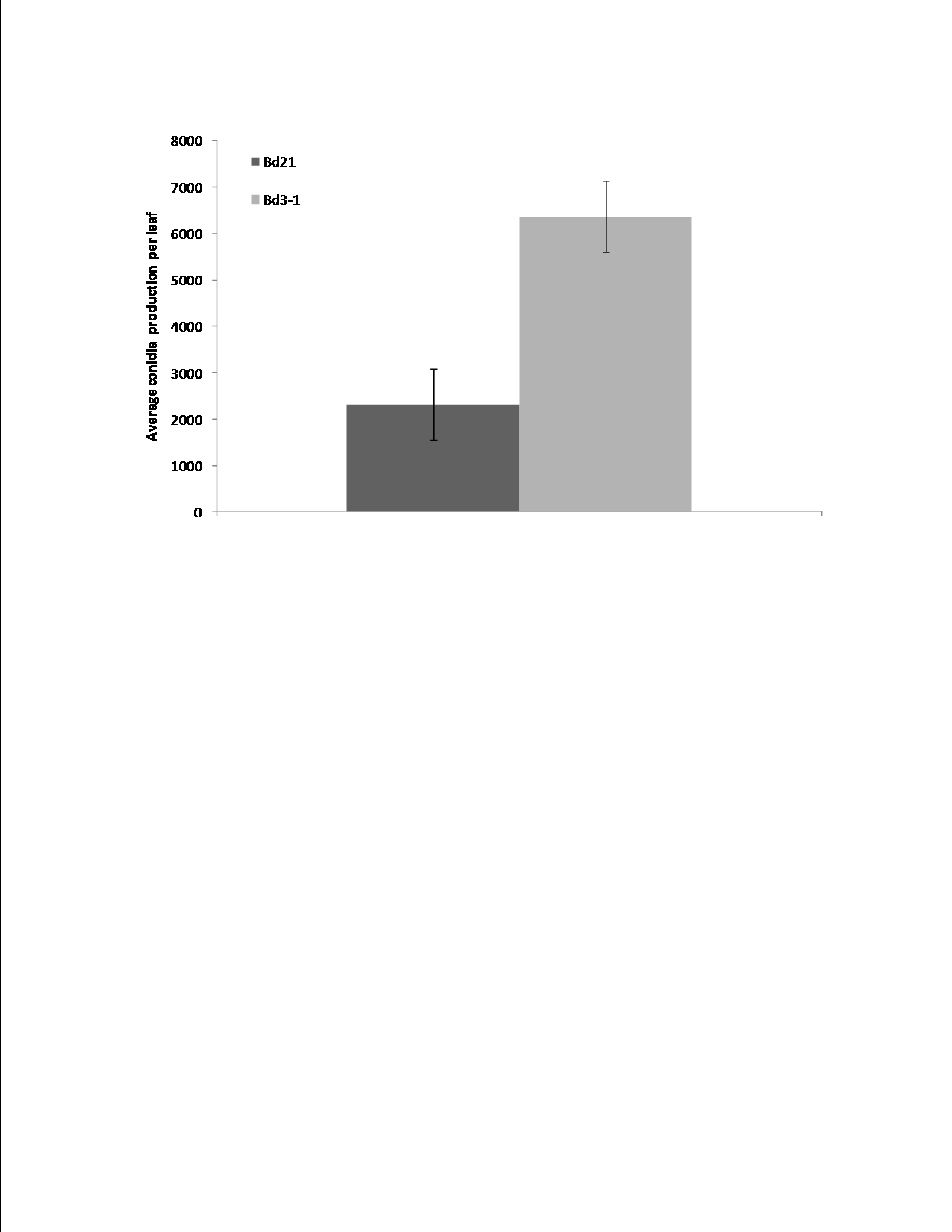

Supplement: Additional file 2 — Comparison of Fg conidial production on Bd21 and Bd3-1 detached leaves. Conidial production following inoculation of Fg UK1 onto Bd21 and Bd3-1 detached leaves, 7 dpi. [file 1471-2229-11-100-S2.TIFF]

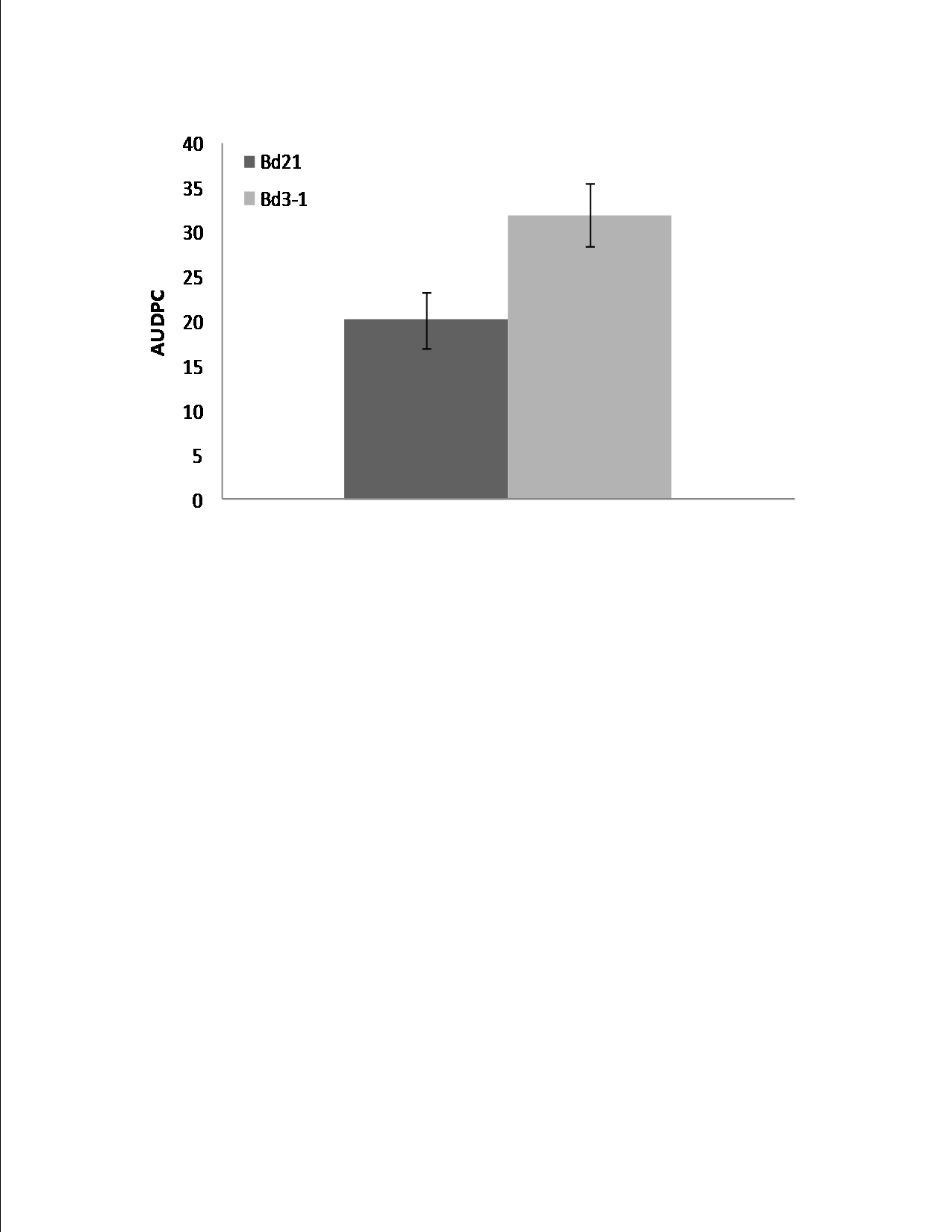

Supplement: Additional file 3 — Comparison of necrotic symptoms development following Fg point inoculation on Bd21 and Bd3-1 spikelets. Area under disease progress curve (AUDPC) for lesions of Bd21 and Bd3-1 spikelets point inoculated with Fg UK1. [file 1471-2229-11-100-S3.TIFF]

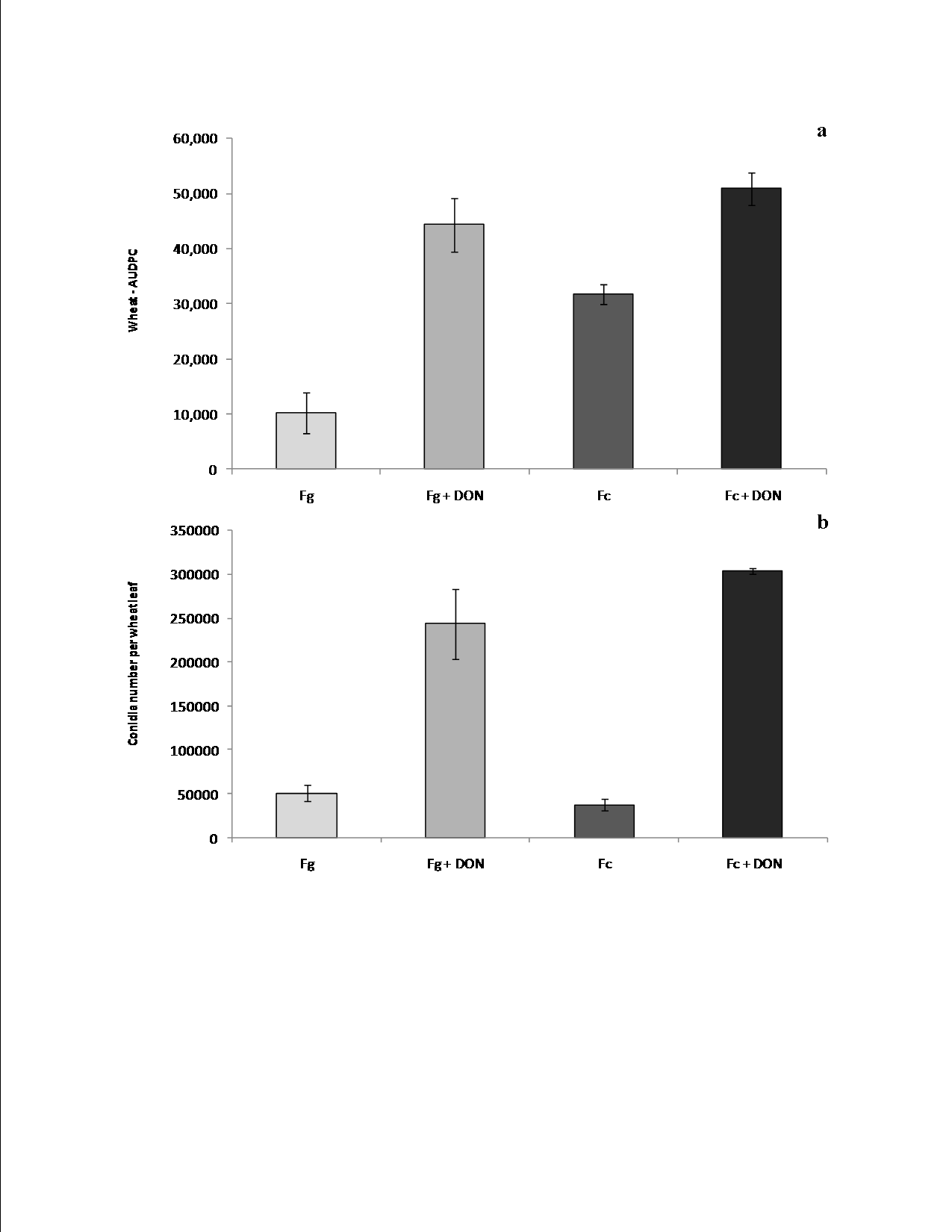

Supplement: Additional file 4 — Effect of DON treatment on detached wheat leaves infected with Fg or Fc. a) Area under disease progress curve (AUDPC) for lesions following wound-inoculation of wheat (cv. Paragon) leaves with Fg UK1 and Fc GFP1 with or without amendment with DON (75 μM). b) Conidial production (6dpi) on leaves of wheat (cv. Paragon) following wound-inoculation with Fg UK1 and Fc GFP1 with or without amendment with DON (75 μM). [file 1471-2229-11-100-S4.TIFF]
